# An Inducible, Ligand-Independent Receptor Activator of NF-κB Gene to Control Osteoclast Differentiation from Monocytic Precursors

**DOI:** 10.1371/journal.pone.0084465

**Published:** 2013-12-27

**Authors:** Cameron W. Rementer, Meiting Wu, Worakanya Buranaphatthana, Hsueh-Ying L. Yang, Marta Scatena, Cecilia M. Giachelli

**Affiliations:** 1 Department of Bioengineering, University of Washington, Seattle, Washington, United States of America; 2 Department of Oral Health Sciences, University of Washington, Seattle, Washington, United States of America; University of California Los Angeles, United States of America

## Abstract

Osteoclasts are bone-resorbing cells that are critical for the normal formation and maintenance of teeth and skeleton. Osteoclast deficiency can contribute to heterotopic ossification (HO), a pathology that is particularly detrimental to the mechanical functions of joints, valves and blood vessels. On the other hand, osteoclast over-activity is a major cause of osteoporosis. A reliable method for controlled generation of osteoclasts would be useful as a potential autologous cell therapy for HO, as well as high-throughput drug screening for anti-osteoporotic drugs. In this report, we describe the development of a cell engineering approach to control monocytic precursor cell differentiation to osteoclasts. Oligomerization of receptor activator of nuclear factor κB (RANK) is known to be essential for osteoclast differentiation from monocyte/macrophage precursors. We engineered a murine monocytic cell line, RAW264.7 to express a fusion protein comprising the intracellular RANK signaling domain and FK506-derived dimerization domains that bind to a small molecule chemical inducer of dimerization (CID). Virally infected cells expressing this fusion protein were treated with CID and dose-dependent induction of tartrate-resistant acid phosphatase activity, as well as multinucleated osteoclast formation were observed. Furthermore, NF-κB signaling was upregulated in a CID-dependent fashion, demonstrating effective RANK intracellular signaling. Functionally CID-induced osteoclasts had robust mineral resorptive activity in both two-dimensional and three-dimensional *in vitro* resorption assays. In addition, the CID-induced osteoclasts have the same life span as native RANKL-induced osteoclasts. Most importantly and crucially, the engineered cells differentiated into osteoclasts that were resistant to the potent osteoclast inhibitor, osteoprotegerin. Taken together, these studies are the first to describe a method for inducible control of monocytic precursor differentiation to osteoclasts that may be useful for future development of an engineered autologous cell therapy as well as high-throughput drug testing systems to treat diseases of osteoclast over-activity that are independent of osteoprotegerin.

## Introduction

Diseases related to osteoclast deficiency as well as osteoclast over-activity have been well described. Heterotopic ossification (HO) refers to abnormal deposition of calcium salts, often taking the form of bone in soft or hard tissues as a result of genetic mutation, trauma or disease [1]. HO can occur as a result of trauma or disease in joints, amputation sites, blood vessels, and heart valves and is frequently found in soldiers wounded by high-energy blasts [2]. There are currently no local or systemic therapies that effectively treat HO, and surgical approaches have had limited efficacy [3], [4]. Radiation therapy is effective when it is delivered to prevent HO, but it is not beneficial once HO is formed [5]. Thus, a new therapy aimed at preventing and/or regressing HO would have enormous health benefits for a wide variety of patients. In HO, osteoblasts and mature bone are observed in calcified lesions, but a paucity of osteoclasts has been noted, consistent with a potential role of osteoclast deficiency in the etiology of this pathology. For example, in a study of explanted calcified aortas containing bone-like lesions, osteoclasts were seen in less than 4% of the samples [6]. Thus, osteoclasts have been proposed as a potential cell therapy to prevent or regress the mineral found in HO [7].

On the other hand, osteoclast over-activity causes osteoporosis, tumor-induced bone loss, and peri-prosthetic osteolysis [8], [9].Current anti-osteoclastic therapies such as bisphosphonates and denosumab are effective, but side effects limit their use. Thus, new therapies continue to be explored using *in vitro* osteoclast resorption assays. These assays require culturing osteoclast precursor cells from bone marrow or human peripheral blood in the presence of two cytokines, macrophage colony stimulating factor (M-CSF) and receptor activator of nuclear factor-κB ligand (RANKL) [10], [11]. This allows the study of the effects of drug candidates on resorption activity of mature osteoclasts [12]. However, isolation and differentiation of rare bone marrow derived monocytic precursors or peripheral blood mononuclear cells into mature osteoclasts is a long and costly process *in vitro*. Moreover, the cytokines that trigger osteoclast differentiation are not only expensive but also have a very short half-life in solution. Clearly, a method that allows for rapid differentiation of osteoclasts from monocytic precursors independent of cytokines could be useful in the development of osteoclast cell therapy as well as high-throughput drug testing systems.

The mechanisms regulating osteoclast differentiation from monocytic precursors have been extensively studied. Two key cytokines, M-CSF and RANKL are necessary and sufficient for osteoclast differentiation and activation. M-CSF induces monocytic precursors in bone marrow to proliferate and the binding of RANKL to its receptor, RANK, drives osteoclast differentiation, fusion, activation and survival. RANK is a type I transmembrane protein originally cloned from dendritic cells [13]. RANK belongs to the tumor necrosis factor receptor (TNFR) superfamily and assembles into functional trimers upon ligand binding. Trimerization triggers downstream NF-κB, MAPK and phosphatidylinositol signaling required for osteoclast differentiation [14], [15]. Osteoprotegerin (OPG) is a potent inhibitor of osteoclast differentiation and survival by acting as a decoy receptor for RANKL [16]. Together, M-CSF and the RANK/RANKL/OPG axis act as major regulators of osteoclast formation and function in the bone.

In the present study, we describe a novel bioengineered system for conditional regulation of osteoclast differentiation from monocytic precursors. This system is based on the chemical inducer of dimerization (CID) technology that has been used extensively to regulate growth and apoptosis of genetically modified cells [17]–[21]. The system generally consists of an intracellular receptor signaling domain linked to a fusion protein (FKBP12) that provides a binding site for a drug called CID [22]–[24]. Since RANK requires trimerization for effective signaling [16], we have engineeered two FKBP12 domains fused to the RANK cytoplasmic domain to ensure successful oligomerization. Our data demonstrate that trimerization/oligomerization of engineered RANK recepter generates fully functional osteoclasts from monocytic precursors. To the best of our knowledge, this is the first use of CID technology to control any type of cellular differentiation, and may be used in the future to develop osteoclast cell therapies or high throughput testing systems for drug discovery.

## Materials and Methods

### Reagents and antibodies

RANKL, OPG and monoclonal anti-human/mouse/rat FKBP12 antibodies were obtained from R&D Systems (Minneapolis, MN), anti-RANK antibody was obtained from Cell Signaling Technology (Danvers, MA), and HRP-conjugated goat-anti-rabbit antibody was obtained from Jackson ImmunoResearch Laboratories, Inc. (West Grove, PA). AP20187 was purchased from ARIAD Pharmaceutics (Cambridge, MA). Leukocyte Acid Phosphatase Assay kit was purchased from Sigma (St Louis, MO). Cathepsin K antibody was purchased from Abcam (Cambridge, MA). Lipofectamine 2000 was purchased from Invitrogen (Carlsbad, CA). Luciferase assay system was obtained from Promega Corporation (Madison, WI). BD BioCoat Osteologic discs were purchased from BD Biosciences (Bedford, MA).

### DNA plasmids

A viral vector, pMGIFM-EGFP-IRES-Myr-F2, containing two copies of the F36V-modified FKBP12 domain, enhanced green fluorescent protein reporter, and a c-Src myristylation domain was a gift from Dr. Blau (University of Washington, Seattle, WA). This construct was used to create vector control cells that expressed just two F36V-modified FKBP12 domains (RAW264.7+F2). The RANK cytoplasmic domain (235–625 a.a.) was amplified by PCR from a mouse RANK cDNA template (OriGene, Rockville, MD). This amplicon was ligated in-frame into the pMGIFM-EGFP-IRES-Myr-F2 vector at the C-terminal end of the second F36V domain to yield pMGIFM-EGFP-IRES-Myr-F2-cRANK. To create the CID-regulatable RANK lentiviral construct (iRANK), the fragment of EGFP-IRES-Myr-F2-cRANK was digested with BamHI (followed by Klenow treatment to blunt the ends) and EcoRI and ligated into pEMlenti vector, a gift from Dr. Murry (University of Washington, Seattle, WA) between BsrGI (followed by Klenow treatment to blunt the ends) and EcoRI.

### Cell culture

RAW264.7 cells were obtained from ATCC (Manassas, VA) and HEK293T cells were obtained from Invitrogen. Cells were cultured in D-MEM medium from Invitrogen (Carlsbad, CA) containing 10% (v/v) heat-inactivated FBS and 100 U/ml pen/strep (Invitrogen) and incubated at 37°C with 5% CO_2_.

### Lentiviral production and transduction

The packaging plasmids pSL3 (vesicular stomatitis virus G envelope), pSL4 (HIV-1 gag/pol packing genes) and pSL5 (rev gene required for HIV-1 envelope protein expression) were a gift from Dr. Murry (University of Washington, Seattle, WA) [25]. The lentiviral vector was packaged in HEK293T cells as previously described [26], [27] with the following modifications. Briefly, a total of 3.3×10^6^ of HEK293T cells were seeded in 10-cm dishes 24 h prior to transfection and the culture medium was changed just before transfection. A total 12 µg plasmid DNA (2.8 µg transfer vector (iRANK), 0.9 µg pSL3, 5.4 µg pSL4 and 2.8 µg pSL5) was used for the transfection of one dish. The precipitate was formed by adding the plasmids to a final volume of 350 µl ddH_2_O and 50 µl of 2 M CaCl_2_, mixing well, then adding 400 µl of 2× HEPES-buffered saline (50 mM HEPES, 280 mM NaCl and 1.5 mM Na_2_HPO_4_). After incubation at room temperature for 15 min, the solution was added to the cultures and the medium replaced after 14–16 h. The media containing virus was collected after another 48 h and filtered through a 0.45-µm filter. The media containing virus was applied to the target cells, RAW264.7 for overnight incubation. Filtered virus solution was stored at −20°C and the second transduction was performed the next day. Transduced RAW264.7 cells were allowed to expand and selected by FACS sorting for GFP expression to obtain ∼92% transduction efficiency.

### Western Blotting

Cell lysates were prepared as previously described [28], and separated on 4–20% SDS-PAGE reducing gels and transferred to PVDF membranes. Membranes were blocked with 5% milk in TBST buffer for 1 h and incubated with monoclonal anti-human/mouse/rat FKBP12 antibody (R&D Systems) at a 1∶1000 dilution for 1 h. HRP-conjugated goat-anti-rabbit secondary antibody (Jackson ImmunoResearch Laboratories, Inc.) was used at a 1∶40,000 dilution. Blots were developed using a SuperSignal West Dura Chemiluminescent Kit (Thermo Scientific) and the signal was captured with CL-XPosure film (Thermo Scientific).

### TRAP activity

Ten thousand cells were plated in 48-well plates and treated with RANKL (40 ng/ml), 0.1% ethanol (EtOH) or varying concentrations of AP20187 (ARIAD Pharmaceutics, Cambridge, MA) and the supplemented media was changed every 2 days for 4 days. Cells were washed twice with PBS and lysed with 60 µl lysis buffer containing 100 mM Na Acetate pH 5.2, 50 mM Na Tartrate pH 4.9 and 2% NP40 and incubated at RT for 10 min. Forty µl of cell extract was combined with 40 µl of reaction buffer (lysis buffer with 2.5 mM N-ASBI-P) in a 96-well plate at 37°C for 30 min, and the reaction was stopped by the addition of 20 µl 0.5 M NaOH [29]. Fluorescence was measured using a microplate reader Safire^2^ (Tecan Group Ltd, Switzerland) with an excitation wavelength of 405 nm and peak emission wavelength of 520 nm.

### TRAP staining

Twenty thousand cells/well were plated in 4-well Lab-Tek™ chamber slides (Nalge Nunc International., Rochester, NY) and treated with vehicle (0.1% EtOH), RANKL (40 ng/ml) or AP20187 (0.1–50 nM) for 4 days. Cells were washed twice with PBS, fixed with 10% buffered formalin for 5 min and subjected to tartrate resistant acid phosphatase (TRAP) staining (Sigma., St Louis, MO) following the manufacturer's instructions. Slides were mounted with Aqua-Mount and images were obtained using an upright microscope (Nikon E800).

### Cathepsin K staining

For Cathepsin K immunohistochemistry, cells were cultured on chamber slides and fixed as described previously (TRAP staining), before blocking endogenous peroxidase with 0.2% H_2_O_2_ (Sigma). Four percent of serum (Vector) was applied to block nonspecific binding. Slides were incubated in a 1∶200 dilution of Cathepsin K primary antibody (Abcam). Normal IgG was used as control. Detection was performed using DAB substrate (Sigma).

### Transfection and Luciferase assays

One hundred thousand cells/well were plated in 48-well plates. The following day, cells were transfected with 0.4 µg total DNA containing an equal amount pBIIX-LUC reporter construct, and a Renilla luciferase construct (pRL) [30] together with 1.5 µl Lipofectamine 2000 (Invitrogen., Carlsbad, CA). Transfection reagents were replaced with fresh serum free medium after 6 h and cells were allowed to recover overnight. Transfected cells were treated with either lipopolysaccharide (LPS) (100 ng/ml), RANKL (40 ng/ml), or AP20187 (20 nM) and cells were harvested at 2 h, 4 h and 6 h. Luciferase activity was measured using a luciferase assay system (Promega Corp.) according to manufacturer's instructions and normalized using the Renilla luciferase.

### Mineral Resorption Assays

RAW264.7+iRANK (2×10^3^ cells/disc) cells were cultured on BD BioCoat Osteologic discs (BD Biosciences, Bedford, MA) in the presence of either RANKL (100 ng/ml) or AP20187 (100 nM) and the supplemented media was changed every 2 days for 10 days. The cells on the discs were removed with 10% bleach. Discs were stained with von Kossa reagent following the manufacturer's instruction (BD Biosciences, Bedford, MA). Resorption pits were visualized using stereomicroscopy (Nikon SMZ1500) and quantified by image analysis.

Calvarial discs were prepared from 4–5 week old C57BL/6 mice. Mice were euthanized by carbon dioxide and the top of the skull was excised. A biopsy punch was used to create 5 mm diameter discs, two from each skull. These were cleaned of adherent tissue, followed by washing twice in phosphate buffered saline (PBS) containing 1% antibiotic/antimycotic. The discs were sonicated twice in 70% EtOH for 10 min. Before use, discs were placed in serum-containing media overnight. RAW264.7+iRANK cells were seeded onto the concave side of each disc and cultured in the presence or absence of AP20187. Media was changed every other day or as needed.

### Confocal Microscopy

Confocal microscopy was used to image live RAW264.7+iRANK cells on calvarial discs. Just before imaging, Hoechst 33258 nuclear stain (Sigma-Aldrich, Inc.) was added to each well at a concentration of 1 µg/ml. A Zeiss 510 META Laser Scanning Microscope (LSM) was used to image the blue fluorescence from the Hoechst stain and GFP expression by the RAW264.7+iRANK cells. Images were processed using Zeiss LSM software.

### Osteoclasts cultured on dentine slices

Dentin slices are an established model for examining osteoclast activity on a physiologically relevant substrate [31]. Human teeth were obtained from local dentists. Teeth were sectioned into 600 µm slices using a Buehler low speed saw equipped with a diamond wafering blade. Dentin slices were cleaned in the same manner as calvarial discs as previously described, and dried until use. RAW264.7+iRANK cells were seeded on dentin slices and cultured in the presence or absence of AP20187. After 10 days, cells were fixed with 10% formalin, and TRAP staining was performed as previously described.

### Osteoclasts cultured on mineralized microporous fibrin scaffolds

Mineralized microporous fibrin scaffolds were prepared as previously described [32]. Briefly, polymethylmethacrylate (PMMA) beads were sintered at 174°C for 22 h before being infiltrated with a solution of fibrinogen and thrombin. After 16 h, the PMMA was dissolved away through acetone washes. Following this, the scaffolds were incubated with a physiological mineralizing solution for 48 h. These scaffolds were seeded with either RAW264.7+iRANK or RAW264.7 cells (2×10^4^ cells/scaffold). The media was supplemented with AP20187 at 50 nM. At days 2, 5, 8 or 11 some of the scaffolds were removed and weighed. They were then fixed in 10% formalin for 2 h before processing for histology. TRAP staining was performed as previously described.

### OPG inhibition study

RAW264.7 and RAW264.7+iRANK cells were plated at 1×10^4^ cells/well in 48-well plates. Six hours after plating, cells were treated with the media containing either 1 nM RANKL or 10 nM AP20187 with increasing concentrations of OPG (RANKL or AP20187 to OPG at 1∶1, 1∶5 and 1∶10 molar ratio). Cells were cultured for 5 days with media replaced once at day 3. Cells were then fixed and TRAP stained and imaged by light microscopy. Multinucleated TRAP-positive osteoclasts (with≥3 nuclei) were quantitated.

### Reverse Transcriptase-PCR

Total RNA was isolated from untransduced RAW264.7, RAW264.7+iRANK, and RAW264+F2. cDNA synthesis was performed using RevertAid First Strand cDNA synthesis kit (Fermentas, Rockford, IL). cDNAs were then used in reverse transcriptase PCR using GoTaq DNA polymerase (Promega, Madison, WI). Primers for RANK were designed to span the region coding the extracellular domain of the molecule, thus amplifying endogenous RANK only. The PCR primers used were: forward 5′-tggacacctggaatgaagaag-3 and reverse 5′-cactcgcagtctgagttcca-3′

### Ethics Statement

Animal work was carried out to minimize animal discomfort by following the Guide for the Care and Use of Laboratory Animals of the National Institutes of Health. This work was approved by the University of Washington Institutional Animal Care and Use Committee (Protocol #2224-04). Discarded human teeth without identifying information meeting the University of Washington definition of “not human subjects” were obtained from local dentists.

### Statistical Analysis

Results are expressed as mean ± SD unless otherwise specified. Significance between groups was determined by ANOVA and p-values less than 0.05 were considered significant.

## Results

**Figure 1:**
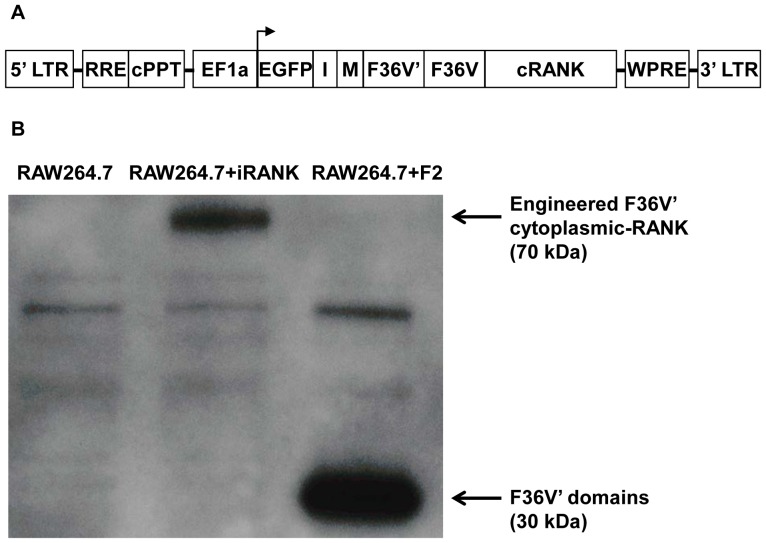
Schematic representation of CID-inducible cytoplasmic RANK (iRANK) lentiviral construct and Western blot detecting construct. A) LTR = long terminal repeat; RRE = rev response element; cPPT = central polypurine tract; EF1a = elongation factor 1-Alpha; EGFP = green fluorescent protein; I = IRES; M = myristoylation; F36V = FKBP12; F36V' = modified FKBP12; cRANK = cytoplasmic domain of RANK; WPRE = WHP posttranscriptional regulatory element. B) Western blot probed with an antibody for FKBP12 showing overexpression of the iRANK construct in RAW264.7+iRANK cells migrating around 70 kDa. Cells transduced just with the F36V' domains (RAW264.7+F2) show a band around 30 kDa. No bands appear in the untransduced RAW264.7 cells.

In order to control RANK signaling and promote differentiation of monocytic precursors to osteoclasts, a fusion construct encoding the RANK receptor intracellular signaling domain and dimerization domains was engineered. We have used an optimized immunophilin analogue, FKBP12 (F36V) that binds specifically to second generation CIDs (e.g. AP1903, AP20187 or AP23510) that are not immunosuppressive and have a markedly reduced affinity for endogenous FKBP12. The cytoplasmic domain of RANK was fused to two F36V domains allowing oligomerization upon the binding of AP20187. Additionally, because the engineered RANK lacks the extracellular domain that normally binds RANKL, this system is RANKL-independent. A schematic representation of the CID-inducible cytoplasmic RANK (iRANK) lentiviral construct is shown in Figure 1. The iRANK construct was transduced into a murine monocytic cell line, RAW264.7 and stably transduced cells were selected by FACS using GFP expression. To determine whether the cells were indeed expressing the CID-inducible receptor fusion proteins, we utilized an antibody directed against FKBP12, which can detect the F36V fusion protein in our constructs due to shared sequence homology. A 70 kDa band consistent with the iRANK fusion protein was observed in the RAW264.7+iRANK cells but not in the non-transduced RAW264.7 cells by Western blot (Figure 1B). As expected, a smaller band around 30 kDa was seen in the cell lysate of cell transduced with the F36V' domains alone (RAW264.7+F2). In addition, Western blotting with an anti-RANK antibody and RT-PCR with endogenous RANK specific primers indicated expression of RANK in all cells at the protein and RNA level, respectively (data not shown).

**Figure 2:**
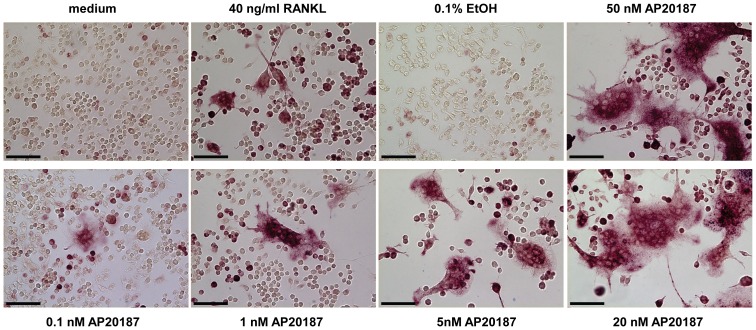
CID-responsiveness of the iRANK construct. RAW264.7+iRANK cells were cultured in medium containing vehicle (EtOH), RANKL, or 0.1–50 nM AP20187 for 4 days and the cells were stained for TRAP. RANKL and AP20187 induced multinucleated TRAP-positive cells were observed (purple staining) (scale bars = 100 µm).

**Figure 3:**
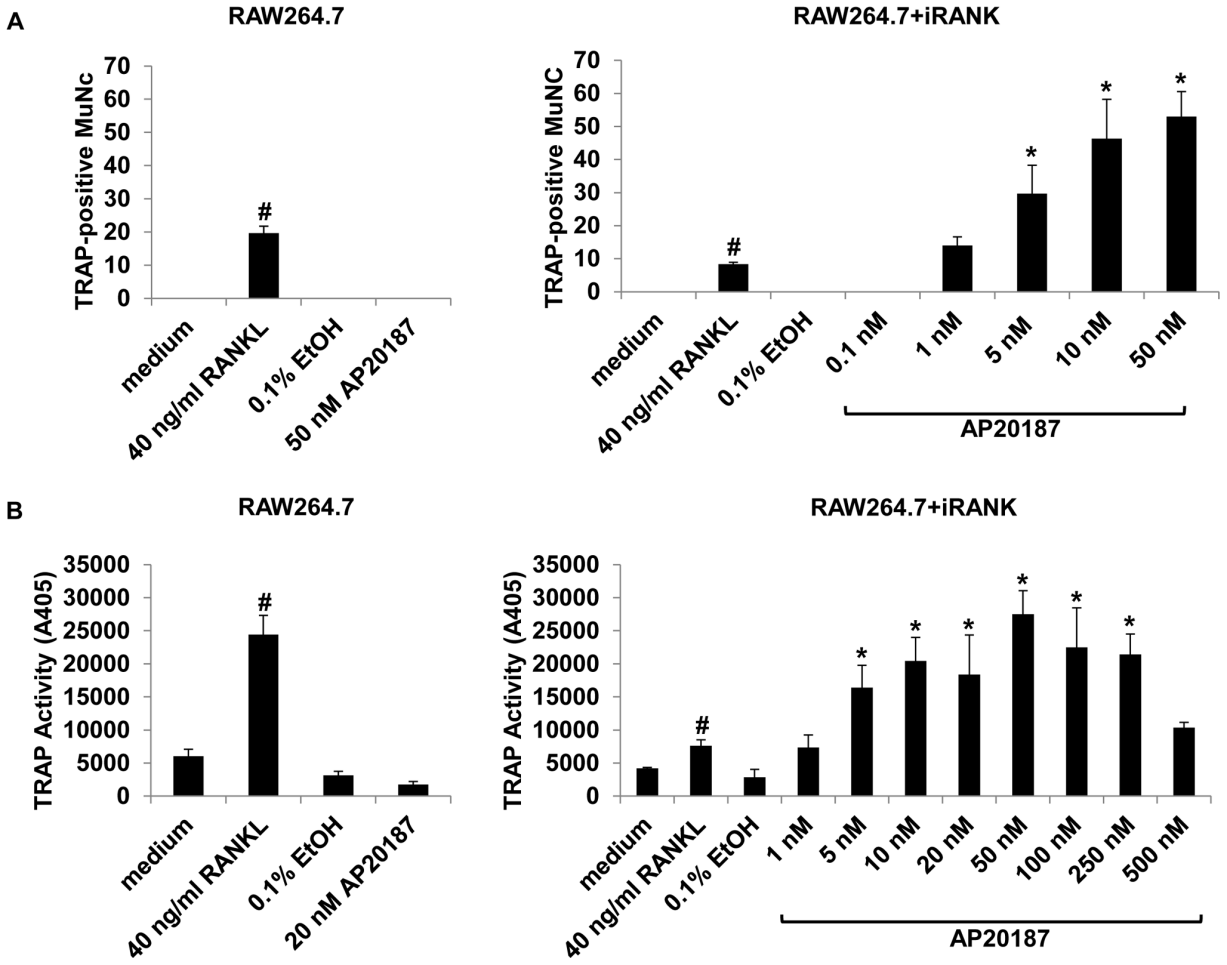
Dose-dependency of AP20187. (A) RAW264.7 and RAW264.7+iRANK cells were cultured in medium alone, or medium containing vehicle (EtOH), RANKL, or 0.1–50 nM AP20187 for 4 days and the cells were stained for TRAP. The number of TRAP-positive multinucleated cells (MuNC) was determined per 4 high power fields of view and averaged over 3 wells. Cells containing more than three nuclei were counted as multinucleated cells. (B) Dose-dependent induction of TRAP activity following RANKL or AP20187 treatment. Left panel: RAW264.7 cells. Right panel: RAW264.7+iRANK cells. The TRAP-activity in RAW264.7+iRANK cells reached a maximum at the 50 nM AP20187 treatment. #p<0.05 compared to medium, *p<0.05 compared to 0.1% ETOH.

To verify CID-responsiveness of the iRANK-transduced cells, RAW264.7+iRANK cells were treated with vehicle alone (EtOH), RANKL, or increasing concentrations of the CID, AP20187. RAW264.7+iRANK cells started to fuse and form multinucleated cells at day 3 with AP20187 treatment and the number of fused multinucleated cells increased with increasing concentrations of AP20187 (Figure 2). Treatment of non-transduced RAW264.7 cells with RANKL induced osteoclast formation as expected, but no osteoclasts were observed in the presence of AP20187 at any concentration tested (data not shown). To confirm that the fused multinucleated cells were osteoclasts, TRAP activity, an important cytochemical marker of osteoclasts was examined. Both RAW264.7 and RAW264.7+iRANK cells were treated with either RANKL or AP20187 for 4 days and stained for TRAP. Robust TRAP-positive multinucleated osteoclast formation was observed in AP20187 treated RAW264.7+iRANK cells after four days, whereas untreated or vehicle (EtOH) treated RAW264.7+iRANK cells showed no osteoclast formation. Treatment of non-transduced RAW264.7 cells with RANKL induced TRAP-positive multinucleated cell formation as expected, but no TRAP-positive multinucleated cells were observed in the presence of AP20187 at any concentration tested (data not shown). Quantitatively, the number of TRAP-positive multinucleated cells induced with the lowest concentration of 1 nM AP20187 in RAW264.7+iRANK was comparable to that induced by 40 ng/ml RANKL in RAW264.7 cells (Figure 3A). Furthermore, TRAP activity in RAW267.4+iRANK cells reached maximal levels at 50 nM of AP20187 and the cells were induced by AP20187 in a dose-dependent manner (Figure 3B). In addition to expressing TRAP, the cells were positive for Cathepsin K, consistent with an osteoclast lineage (data not shown).

**Figure 4:**
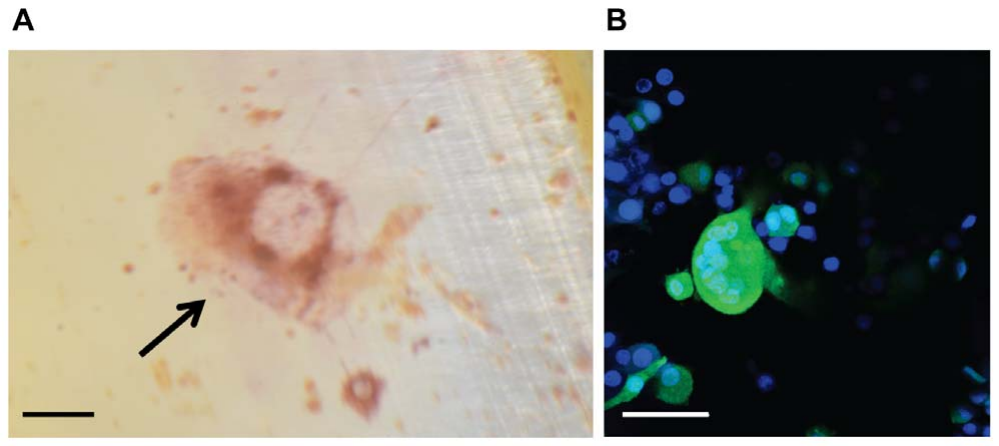
CID induced osteoclasts formed on native mineralized substrates. (A) TRAP-positive osteoclasts on human dentin slices. Dentin slices created from human teeth were seeded with RAW264.7+iRANK cells and cultured in the presence of AP20187 for 9 days. The slides were fixed and stained for TRAP and multinucleated TRAP-positive cells were shown in purple (arrow) (scale bar = 100 µm). (B) Confocal micrograph of multinucleated osteoclasts on mouse calvarial disc. Calvarial discs were seeded with RAW264.7+iRANK cells and osteoclast formation was induced by the addition of AP20187. Cells were imaged by confocal microscope; blue fluorescence indicates nuclei by Hoechst stain, while green fluorescence indicates GFP expression in RAW264.7+iRANK cells (scale bar = 50 µm).

To determine whether CID treatment could induce osteoclast formation in iRANK transduced cells on native mineralized surfaces, human dentin slices and mouse calvarial discs were investigated. RAW264.7+iRANK cells were cultured and induced with AP20187 on dentine slices prepared from human teeth. TRAP-positive multinucleated cells were observed in response to AP20187 treatment (Figure 4A) but not in the absence of CID (data not shown). Likewise, AP20187 treatment induced osteoclast (multinucleated cells with three or more nuclei as detected by DAPI) formation on calvarial discs prepared from mice (Figure 4B).

**Figure 5:**
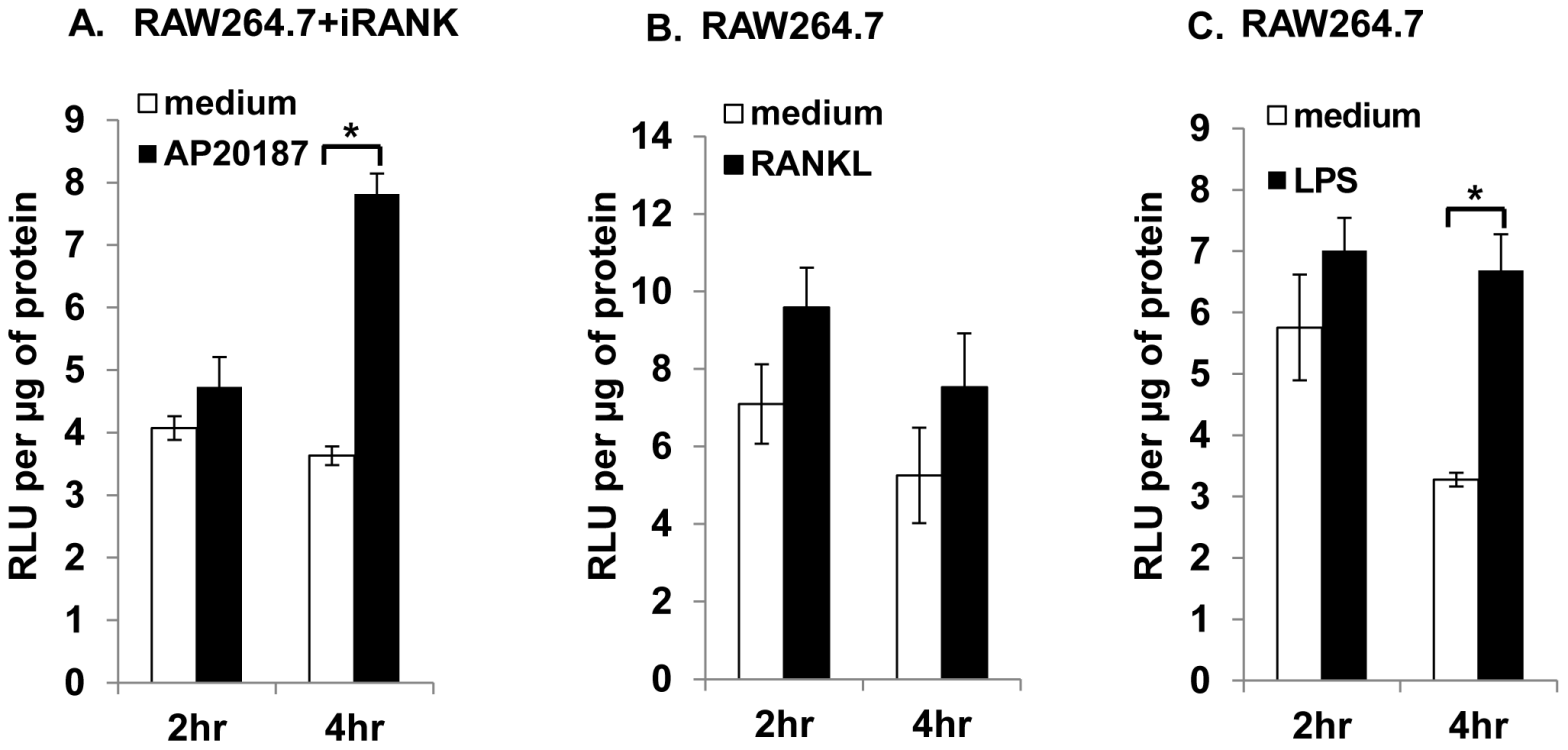
NF-κB signaling. NF-κB dependent signaling in engineered osteoclasts. RAW264.7 and RAW264.7+iRANK cells were transiently transfected with a luciferase reporter construct containing NF-κB sites derived from Igκ promoter driving the luciferase gene and a Renilla luciferase construct as the internal control. NF-κB activation was measured in RAW264.7+iRANK cells stimulated with AP20187 (A), and RAW264.7 cells stimulated with RANKL (B) or LPS (C) for 2 and 4 h. Data are average relative light units (RLU) per µg protein +/− SD. *p<0.05.

Next, NF-κB signaling and osteoclast mineral resorption functions were examined. NF-κB is the major signaling pathway activated by RANK in osteoclasts and the activity of NF-κB transcription factor is crucial for osteoclastogenesis [33], [34]. To examine NF-κB activation by AP20187, both RAW264.7 and RAW264.7+iRANK cells were transiently transfected with two plasmids, a luciferase reporter construct containing NF-κB sites derived from Igκ promoter driving the luciferase gene and a Renilla luciferase construct as the internal control. The transfected cells were then stimulated with either AP20187, RANKL, or LPS (a RANK independent NF-κB inducing agent) and luciferase activity was monitored after 2 and 4 h. The activation of NF-κB by AP20187 in RAW264.7+iRANK cells or by RANKL and LPS in RAW264.7 cells was observed as early as at 2 h after stimulation. At 4 h, AP20187-stimulated NF-κB activity in RAW264.7+iRANK cells (7.8 RLU/µg protein) was comparable to RANKL- (7.3 RLU/µg protein) and LPS- (6.7 RLU/µg of protein) stimulated NF-κB activity in RAW264.7 cells (Figure 5). This result suggests that the transduced iRANK construct can mediate NF-κB signaling in response to AP20187 treatment in RAW264.7 cells.

**Figure 6:**
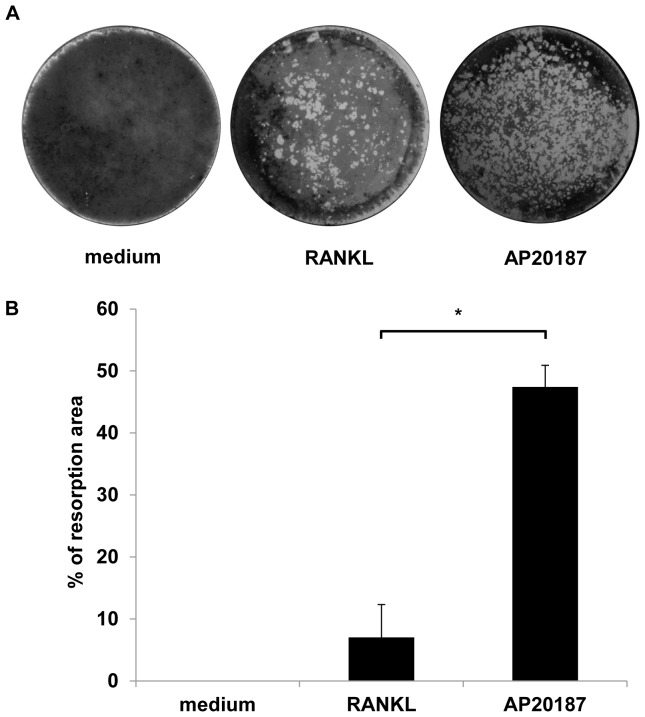
CID induced osteoclasts resorbed a two-dimensional mineralized substrate. (A) RAW264.7+iRANK cells were treated with either RANKL (100 ng/ml) or AP20187 (100 nM) on Osteologic discs. After 10 days, resorption lacunae were visualized by von Kossa staining. (B) The percent resorbed area per disc was measured and analyzed using ImageJ. Three experiments were averaged. Y-axis shows the % resorption per disc. *p<0.05.

To quantify the two-dimensional mineral resorptive properties of AP20187-induced osteoclasts, RAW264.7+iRANK cells were cultured directly on Osteologic discs (BD Biosciences) in the presence of either 100 ng/ml RANKL or 100 nM AP20187 for 10 days. These discs feature a calcium phosphate mineral coating, and can be used to measure mineral resorption. Resorption pits were visualized using von Kossa staining and imaged using a stereo microscope (Figure 6A). The images were analyzed using ImageJ program and the resorption area was quantified as a percent of the whole disc. The resorbed area in AP20187 treated RAW264.7+iRANK cells (∼47%) was significantly higher than the cells treated with RANKL alone (∼7%) (Figure 6B). This is likely due to the high levels of iRANK construct in the cells compared to endogenous RANK, since the cells were sorted to enrich for iRANK expressing cells.

**Figure 7:**
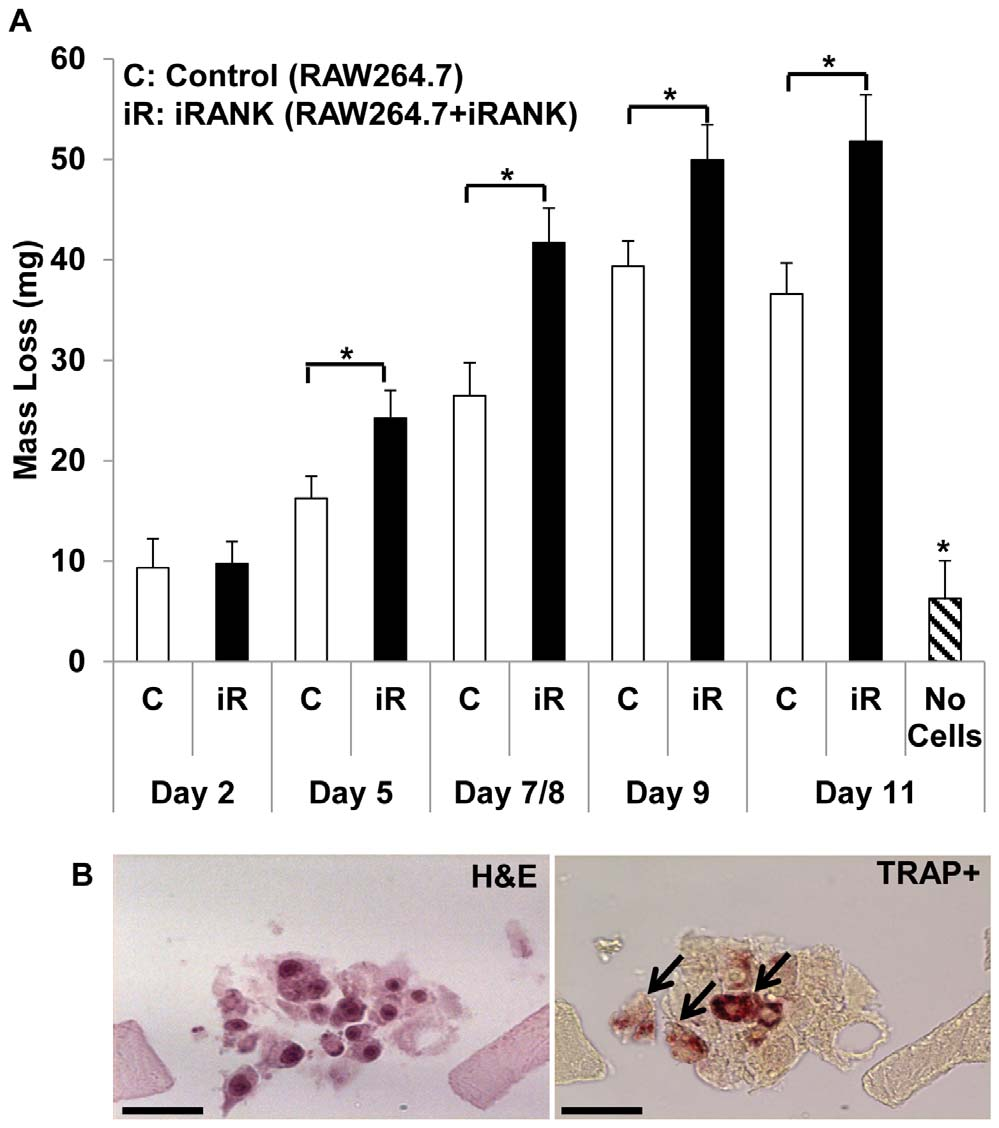
CID induced osteoclasts resorbed a three-dimensional mineralized substrate. (A) Mineralized fibrin scaffolds were seeded with control RAW264.7 cells (white bars) or iRANK transduced RAW264.7 cells (black bars), or no cells (hatched bar). The scaffolds were weighed at various time points (days 2, 5, 8 and 11). The scaffolds without cells were incubated in media for 11 days. Mass loss was calculated by subtracting the final mass from the initial mass. *p <0.05. (B) RAW264.7+iRANK were cultured with AP20187 in the fibrin scaffolds for 8 days. H&E staining (left panel) and adjacent TRAP staining (right panel) indicate the differentiation of osteoclasts within the scaffolds (arrows) (scale bars = 50 µm).

AP20187-induced osteoclasts could also resorb a mineralized three-dimensional matrix in a CID dependent manner. Microporous fibrin scaffolds were generated using sphere templating technology as previously described [32]. Scaffolds had a pore size of 200–250 µm and were mineralized with a physiological mineralizing solution for 48 h, resulting in a calcium content of 34.8±5.6 µg calcium/mg dry scaffold weight. RAW264.7+iRANK cells or unmanipulated RAW264.7 cells were seeded on the scaffolds at a density of 2×10^4^ cells/scaffold. The cells were cultured in media containing 50 nM AP20187. Scaffolds were removed and weighed at various time points (days 2, 5, 8, and 11) following cell seeding. We observed a significant decrease in the weights of the scaffolds seeded with the engineered osteoclasts compared to parental monocytes by day 11. Weight loss was minimal in scaffolds that did not receive cells at day 11 (Figure 7A). When the scaffolds were examined by histology, multinucleated cells were observed by H&E staining within the scaffolds seeded with RAW264.7+iRANK cells, and all of these stained positively for TRAP (Figure 7B), whereas no multinucleated TRAP-positive cells were seen in scaffolds seeded with RAW264.7 cells (data not shown).

**Figure 8:**
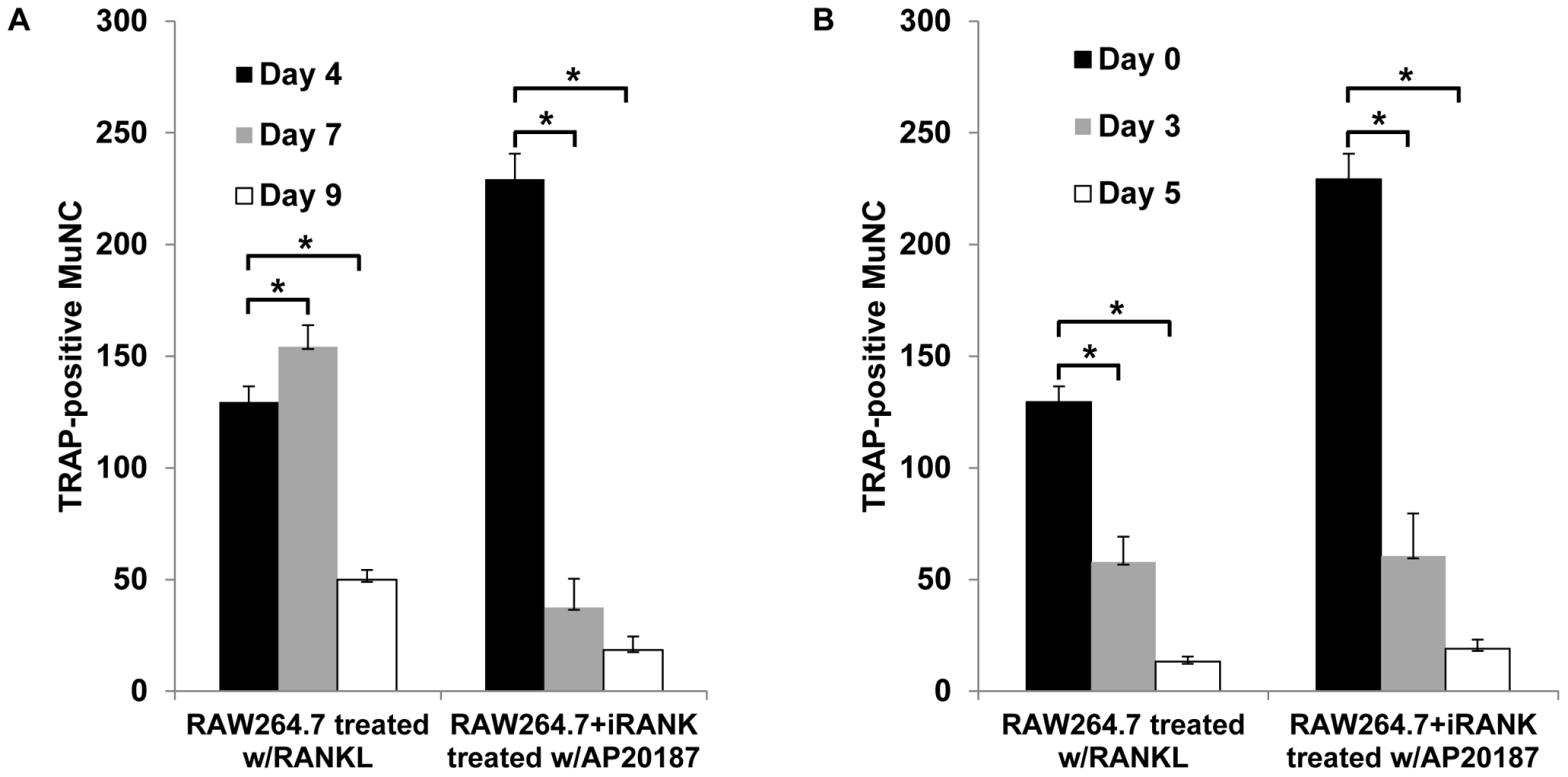
Cell survival study. RAW264.7 cells and RAW264.7+iRANK cells were treated with either RANKL (40 ng/ml) or AP20187 (50 nM) for 4 days to allow osteoclasts to form. The supplemented media was removed, and cells were cultured for additional 0, 3 or 5 days in the presence (A) or absence (B) of inducers, and the number of TRAP-positive multinucleated cells (MuNC) per well was counted and averaged over 4 wells. *p<0.05.

The time course of accumulation of osteoclasts following continuous RANKL or CID treatment of RAW264.7 and RAW264.7+iRANK cells, respectively, is shown in Figure 8A. In RANKL-treated RAW264.7 cells, the number of osteoclasts increased with time and attained a maximum at day 7 followed by a large decline in osteoclast number by day 9. In contrast, CID-treated RAW264.7+iRANK cells achieved maximal osteoclast numbers by day 4, followed by large declines at days 7 and 9. These data suggested a limited life span of the differentiated osteoclasts even in the continued presence of inducing agent. To better examine the life span of RANKL or CID induced osteoclasts, a cell survival study following drug withdrawal from pre-formed osteoclasts was performed (Figure 8B). RAW264.7 and RAW264.7+iRANK cells were treated with 40 ng/ml RANKL or 50 nM AP20187, respectively, for 4 days after which the drug was withdrawn (day 0) and the cells were cultured in media alone for another 3 or 5 days. The cells were fixed, TRAP stained, and multinucleated TRAP-positive osteoclasts were counted. At day 3 of drug withdrawal, the number of osteoclasts decreased to 44% of day 0 values in RAW264.7 cells and to 26% of day 0 values in RAW264.7+iRANK cells. By day 5 of drug withdrawal, there were only ∼10% osteoclasts surviving in RAW264.7 cells and ∼8% in RAW264.7+iRANK cells. These data suggest that regardless of inducing agent, the osteoclasts had a similar lifespan of about 5 days *in vitro*.

**Figure 9:**
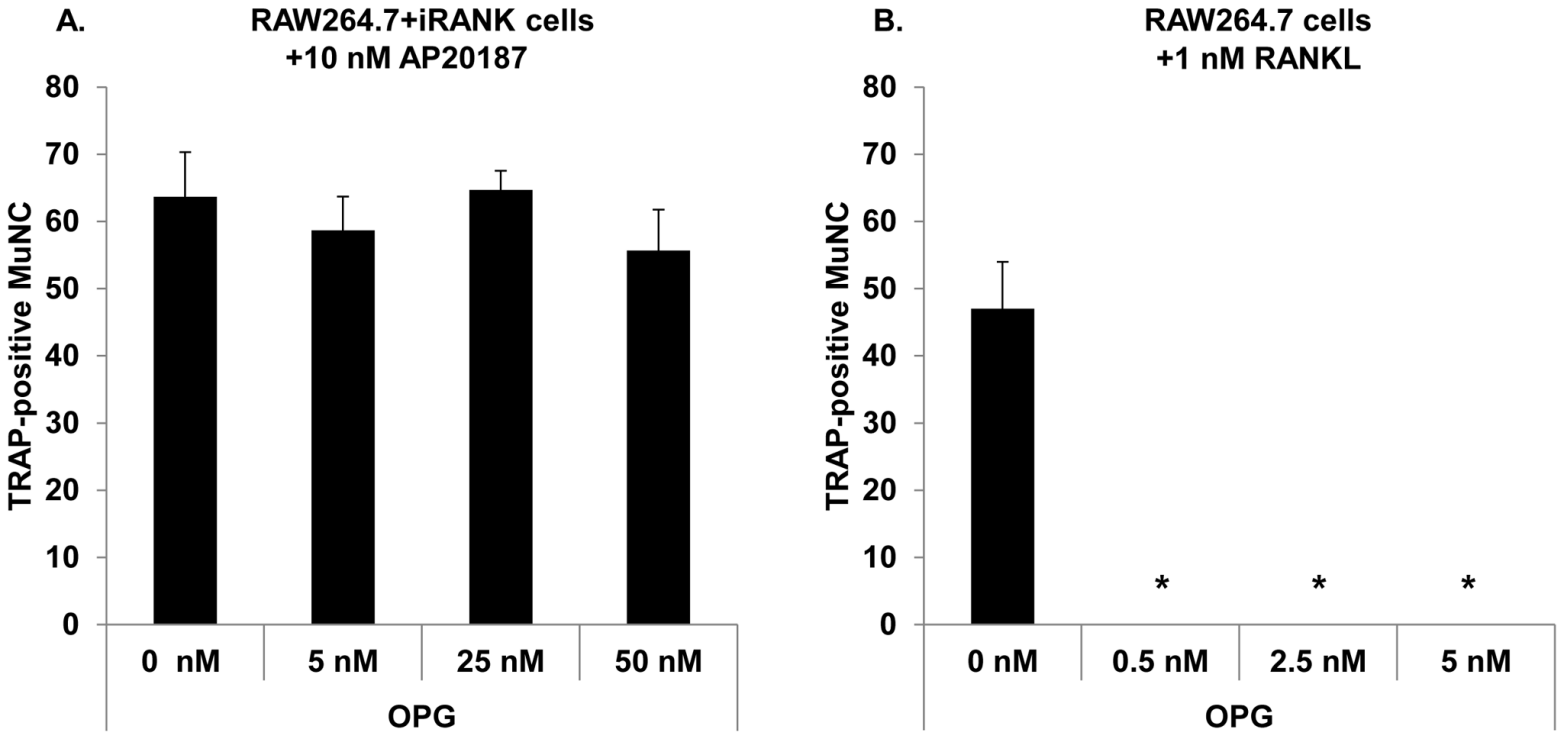
CID induced osteoclastogenesis in RAW264.7+iRANK cells is OPG-independent. TRAP-positive multinucleated cells (MuNC) after treatment of RAW264.7+iRANK cells with 10 nM AP20187 (A) or RAW264.7 cells with 1 nM RANKL (B) in the presence of increasing concentrations of OPG. TRAP-positive multinucleated cells (MuNC) were counted over 4 high power fields of view and averaged over 3 wells. *p<0.05 compared to 0 nM OPG.

Finally, to determine if AP20187-induced osteoclasts were resistant to the osteoclast differentiation inhibitor, OPG, RAW264.7+iRANK cells were treated with AP20187 in the presence of increasing concentrations of OPG for 5 days. As shown in Figure 9A, the total number of multinucleated TRAP-positive cells per well was unchanged even in the presence of the highest concentration of OPG (50 nM). In contrast, RANKL-mediated osteoclastogenesis was completely inhibited by OPG even at the lowest concentration (0.5 nM) (Figure 9).

## Discussion

In this study, we applied CID technology using a RANK fusion receptor to control monocytic precursor differentiation into osteoclasts. The engineered RAW264.7+iRANK cells differentiated into TRAP and Cathepsin K positive, multinucleated osteoclasts in a dose dependent manner in response to CID treatment. In addition, appropriate activation of the NF-κB signaling pathway was observed following CID treatment in these cells. The engineered osteoclasts showed robust mineral and matrix resorptive activity in two and three-dimensional model systems. Futhermore, CID-induced osteoclasts had a similar life span compared to RANKL-induced osteoclasts, and lifespan was not altered by CID treatment. Finally, CID-induced osteoclast differentiation occurred even in the presence of high concentrations of the natural RANK antagonist, OPG.

Although CID technology has been used as a proliferation or death switch for genetically engineering cells for over a decade [17], our studies are the first to successfully apply this technology to control monocyte differentiation to functional osteoclasts. A previous report attempted the use of one dimerization domain fused to the cytoplasmic domain of RANK to induce RANK dimerization upon binding of the CID, AP20187 [35]. However, use of this construct to induce simple dimerization of RANK failed to induce complete multinucleated osteoclast formation, and both bone resorption activity and expression of osteoclast differentiation markers were much lower than in RANKL-stimulated cells. In contrast, we utilized two dimerization domains fused to cytoplasmic RANK receptor (iRANK construct) that could allow for RANK trimerization or higher order oligomerization following CID binding. As we have shown, the iRANK construct in the presence of CID triggered RAW264.7 cell differentiation into fully functional, multinucleated osteoclasts with greater bone resorption activity than RANKL-induced cells. In addition, the iRANK engineered cells differentiated into osteoclasts even in the presence of the potent osteoclast inhibitor, OPG. It is interesting to note that decreased responsiveness of the RAW264.7+iRANK cells to RANKL was observed in osteoclastogenesis assays. Since the iRANK construct was targeted to the plasma membrane by a myristoylation sequence, it is possible that overexpression of the iRANK fusion protein could lead to sequestration of factors required for downstream RANK signaling, such as TRAF6 or GAB2. Further experiments are necessary to test this hypothesis.

In the three dimensional mineralized fibrin scaffolds, some mass loss was seen in the control conditions. RAW264.7 cells are a murine monocytic cell line with macrophage-like properties, and monocyte-derived macrophages can secrete plasminogen activator thereby generating plasmin in serum-containing media, as well as fibrin-degrading MMPs, and thus promote fibrinolysis. Thus it is not surprising that unmanipulated RAW264.7 cells were able to breakdown the mineralized fibrin scaffold to some extent. However, the scaffolds seeded with RAW264.7 cells stimulated with the chemical dimerizer, AP20187, had a significantly larger mass loss than the control group. This suggests that there was enhanced mass loss due to the presence of induced osteoclasts.

Our studies provide proof of principle for CID-controlled osteoclast formation from bioengineered monocytic precursors. One potential application for our system would be the treatment of abnormal calcium deposits using an autologous cell therapy. Bone marrow-derived osteoclasts were first reported to have the ability to reduce the mineral content in calcified aortic elastin without degrading the elastin matrix *in vitro*, and thus were suggested as a potential cell therapy for valve disease and other forms of ectopic calcification [7]. However, the idea of using autologous bone marrow-derived osteoclasts as a therapy for ectopic calcification is limited because of the presence of osteoclast inhibitors, like OPG. OPG is up-regulated early in disease progression in valve tissue [36] and in serum. The bioengineered system developed here could overcome this limitation, since osteoclast differentiation of precursors to osteoclasts is controlled by CID-regulated trimerization of the iRANK construct, and thus cannot be inhibited by OPG. Another limitation for using native osteoclasts induced by RANKL as therapy is that the precursor cells must be differentiated into osteoclasts *in vitro* prior to delivery as administering RANKL to initiate osteoclastogenesis is not feasible *in vivo*. Fully differentiated osteoclasts are large and multinucleated which makes them fragile and difficult to deliver, making pre-differentiating osteoclasts for a cell therapy an unviable approach. Our system would allow for the activation of osteoclastogenesis *in vivo* by a small molecule CID. A cell therapy for treating abnormal calcification would involve first delivering mononuclear precursor cells to the desired site, followed by initiating the differentiation of osteoclasts *in situ* by the small molecule CID. This method would overcome the difficulties associated with delivering terminally differentiated osteoclasts to sites of abnormal calcification.

A second application for our technology is high-throughput drug screening. Mature osteoclasts are routinely used *in vitro* as a drug screening tool for discovery of new anti-resorptive therapeutics [12]. However, this system is costly because it requires the use of monocytic precursors from either bone marrow or peripheral blood and the addition of two cytokines, RANKL and M-CSF for osteoclast differentiation. Using our system, differentiation of iRANK engineered osteoclasts was independent of RANKL and M-CSF, and the cells were able to differentiate to functional osteoclasts with as low as 1 nM of CID. Denosumab is a human recombinant monoclonal antibody approved to treat postmenopausal osteoporosis. Its mechanism of action mimics OPG by binding to RANKL, which inhibits osteoclast formation and limits bone resorption. However, denosumab can cause serious side effects including osteonecrosis of the jaws [37], [38] and unusual subtrochanteric fractures similar to bisphosphonate-associated atypical femur fractures [39]. Thus, new categories of drugs that act through other mechanisms are needed for those patients who cannot tolerate existing treatments. The system we developed cannot be inhibited by OPG, which may allow for the identification of new drugs that inhibit osteoclasts through alternative pathways.

In conclusion, we have engineered monocytic precursors to differentiate into osteoclasts under the control of the CID, AP20187. This differentiation is independent of RANKL and M-CSF, and it is also resistant to OPG. When combined with autologous precursors, this system could be used to develop a local cell-based therapy to treat or prevent ectopic calcification. In addition, this system could be used to robustly and cost-efficiently generate osteoclasts for high throughput drug testing, and could facilitate discovery of new therapeutic agents against diseases of osteoclast over-activity that are independent of OPG. Future studies will be needed to move the system to human bone marrow or peripheral blood monocytic precursors for developing autologous cell therapy to treat or prevent ectopic calcification.
